# The Application of Complementary and Alternative Medicine in Polycystic Ovary Syndrome Infertility

**DOI:** 10.1155/2022/5076306

**Published:** 2022-10-07

**Authors:** Yu-Qian Shi, Yi Wang, Xi-Ting Zhu, Rui-Yang Yin, Yi-Fu Ma, Han Han, Yan-Hua Han, Yue-Hui Zhang

**Affiliations:** ^1^Heilongjiang University of Chinese Medicine, Harbin, China; ^2^The First Clinical Hospital affiliated to Harbin Medical University, Harbin, China; ^3^Department of Obstetrics and Gynecology, Key Laboratory and Unit of Infertility in Chinese Medicine, First Affiliated Hospital, Heilongjiang University of Chinese Medicine, Harbin, China

## Abstract

Polycystic ovary syndrome (PCOS) is a lifelong reproductive endocrine disease, which is the most common cause of anovular infertility. Modern medicine mainly treats infertile patients with PCOS by improving living habits, ovulation induction therapy, and assisted reproductive technology (ART), but the effect is not satisfied. Complementary alternative medicine (CAM) has conspicuous advantages in the treatment of PCOS infertility due to its good clinical efficacy, wide mechanism of action, and no obvious adverse reactions, but its safety and effectiveness in the treatment of PCOS infertility have not been proved. Based on the existing clinical and experimental studies, this paper looks for the therapeutic effect and the mechanism behind it, and explores the safety and effectiveness of its treatment in PCOS infertility, in order to provide reference for future clinical treatment and experimental research.

## 1. Introduction

PCOS is a life-long reproductive endocrine disease characterized by anovulatory, hyperandrogenism, and polycystic ovary. It is one of the most common causes of infertility in women of childbearing age. It is usually associated with reproductive complications (irregular menstruation, ovulation dysfunction, and pregnancy complications), metabolic disorders (type 2 diabetes and cardiovascular disease), and even psychological risk factors [1–4]. Depending on the population investigated and the diagnostic criteria used, the prevalence of PCOS ranges from 6% to 15% and approximately 75% of women with PCOS suffer from infertility due to ovulation disorders, which makes infertility an urgent problem for PCOS patients [5–7]. Overexpression of proinflammatory factors and intense oxidative stress (OS) in PCOS patients inhibits follicle stimulating hormone (FSH) and luteinizing hormone (LH) receptor expression, leading to oocyte dysplasia and eventually infertility [8, 9]. For infertile patients with PCOS who have fertility needs, first-line and second-line treatment such as lifestyle adjustment and ovulation induction therapy are usually preferred. For patients with ineffective treatment, they will eventually choose clinical third-line treatment to assist reproductive technology in order to achieve pregnancy. Due to hormone and metabolic problems in PCOS patients receiving ovulation induction, ART process is not good and accompanied by serious adverse reactions, it is necessary to find an alternative therapy to supplement or replace conventional western medicine treatment, in order to solve the poor efficacy of conventional western medicine treatment, adverse reactions, and other drawbacks. CAM refers to a group of medical systems used in combination with or in place of traditional medicine. According to the National Center for Complementary and Integrative Health, CAM models are divided into three categories: natural products, mind-body practices, and other complementary health approaches, and the specific classification varies from socio-culturalbackground [10, 11]. According to international studies, 29–91% of infertile women are using CAM techniques [12–15]. Studies have shown that the use of CAM methods can significantly improve sex hormone levels, relieve IR, reduce weight, regulate mood, and improve ovulation in infertile patients with PCOS [16]. CAM is usually safe for physicians to prescribe on a patient-by-patient basis, but many treatment have contraindications. Since there is insufficient evidence on the safety and efficacy of CAM techniques in the treatment of PCOS, it is necessary to investigate the efficacy and safety of CAM techniques in the treatment of infertile patients with PCOS. All of the studies are listed in Table 1.

## 2. Chinese Medicine Treatment of PCOS Infertility

Traditional Chinese medicine (TCM) is a CAM model, which is guided by the theory of yin-yang and five elements, five viscera, and six fu organs, qi, blood, and fluid, and takes syndrome differentiation and treatment as the treatment principle, and provides individualized diagnosis and treatment for infertility patients with PCOS. TCM diagnosis and treatment is rooted in China's profound traditional cultural background, summarizes and sublimation of more than 5000 years of people's practical experience, combined with modern medical theory of medical diagnosis and treatment. TCM is not limited to improving “symptoms,” but emphasizes the human body, society, and nature as a whole “holistic view,” through a variety of ways to treat patients with physiological and psychological “disease.” TCM has been spread to 196 countries and has achieved satisfactory results in more than one-third of the world's population. Chinese herbal medicine (CHM) is the most distinctive treatment of TCM, which is divided into monomer and compound. Due to its obvious therapeutic effect and high safety, it is widely used in the treatment of infertile patients with PCOS [17]. TCM theory believes that the pathogenesis of PCOS infertility is due to deficiency to excess kidney deficiency, resulting in abnormal liver and spleen function and pathological products such as qi stagnation, phlegm dampness, and blood stasis [18]. In the theory of TCM, kidney contains kidney essence, kidney essence is the key material to nourish the human body to promote the development of reproductive function; it is the most fundamental nutrients in the human body. Therefore, most of the TCMs for PCOS infertility are kidney-tonifying, supplemented by qi-expelling, phlegm-resolving, blood-activating, and stasis-resolving therapies, with good clinical results. Therefore, the majority of scholars have carried out a large number of in vitro and in vivo experiments on TCM, studies have shown that TCM by regulating sex hormone levels, improves IR and promotes follicle development [19–22]. We have listed some RCTs in Table 2.

### 2.1. Clinical Observation of Chinese Medicine in the Treatment of PCOS Infertility

#### 2.1.1. Clinical Observation of Chinese Medicine Monomer in the Treatment of PCOS with Infertility

*(1) Clinical Application of Berberine*. Berberine (BBR), an iso quinoline alkaloid, is widely found in many plants of the Berberaceae family, such as Rhizoma Coptidis and Cortex Phellodendri. BBR was initially used in gastrointestinal infections such as diarrhea because of its excellent antibacterial effect. But in recent years, with the deepening of research, it has been found to be effective in improving IR, lowering androgen levels, and improving glucolipid metabolism, especially in PCOS patients with IR (IR) [23]. BBR can improve insulin sensitivity by regulating the signaling pathway of mTOR-IRS1 in patients to achieve the therapeutic effect of PCOS treatment [24]. For infertile patients with PCOS receiving assisted reproduction, BBR combined with ovulation induction drugs such as cyproterone acetate (CPA), clomiphene citrate (CC), and letrozole (LET) can improve the ovulation induction effect and reduce the incidence of adverse reactions, thus BBR has a high potential research value [25, 26]. However, this conclusion is controversial. Wu et al. found that the pregnancy and ovulation rates of BBR combined with LET in the observation group were similar to those of the control group using BBR alone, which could not indicate that BBR improves pregnancy and ovulation rates in PCOS patients [27]. Further studies are needed to comprehensively evaluate the effect and mechanism of BBR in improving reproductive function in the future.

*(2) Clinical Application of Cryptotanshinone*. Cryptotanshinone (CRY), as the main lipid-soluble component of Salvia miltiorrhiza, has various pharmacological effects such as antibacterial, anti-inflammatory, antioxidant and so on [28]. Modern pharmacological studies have shown that CRY has a regulatory effect on the reproductive endocrine of ovarian organs and can significantly reduce serum androgen levels in PCOS patients [29, 30]. In an experiment using dehydroepiandrosterone to induce PCOS in a rat model, CRY was shown to improve PCO status, regulate the estrous cycle, and reduce testosterone (T), LH, and androstenedione [31]. Yang et al. found that CRY can regulate sex hormone disorder and promote follicle development in PCOS by inhibiting the expression of HMGB1, TLR4, and NF-*κ*B/p65 in ovarian tissue and reducing the level of inflammatory factors such as TNF-*α* [32].

*(3) Clinical Applications of Quercetin*. Quercetin (QUE) is a phytoestrogen commonly found in herbal medicine and has antioxidant, anti-inflammatory, immunoprotective, and even anticancer effects. The most important features of QUE are its estradiol-like structure and phytoestrogen activity, which can improve the clinical symptoms of obesity, infertility, and sex hormone disorders in PCOS [33]. Rezvan et al. have confirmed that QUE can increase adiponectin levels and reduce homeostasis model assessment of insulin resistance (HOMA-IR), T, LH, and insulin levels in PCOS patients [34], which is consistent with the results of Khorshidi [35] et al. The rat model suggests that QUE not only regulates the level of glucolipid metabolism and improves the level of sex hormones, but also has powerful antioxidant ability to improve ovarian PCO status [36].

#### 2.1.2. Clinical Application of Commonly Used Compound Prescriptions

*(1) Clinical Application of Liu Wei Di Huang Prescription*. Liu Wei Di Huang Prescription (LDP, the main dosage form is decoction or pill) is a well-known formula for tonifying kidney yin, which was first recorded in Qian Yi's “Key to Therapeutics of Children's Diseases” in A.D.1114. LDP is composed of six Chinese medicines: *Rehmanniae Radix, Corni Fructus, Dioscoreae Rhizoma, Poria, Alismatis Rhizoma,* and *Moutan Cortex*, which have anti-inflammatory and antioxidant properties and can improve IR and HA [37]. LWP can regulate sex hormone levels and promote ovulation, so it is widely used in the treatment of infertility [38], PCOS [39], premature aging [40], diabetes [41] and other diseases.

*(2) Clinical Application of Gui Zhi Fu Ling Pill*. Gui Zhi Fu Ling Pill (GFP) is a classic formula for activating blood circulation and removing blood stasis, which was first published in Zhang Zhongjing's “Synopsis of Golden Chamber - Women's Chapter” in the Eastern Han Dynasty [42]. GFP is composed of *Cinnamomum cassia Presl, Poria*, *Paeonia lactiflora Pall.*, *Moutan Cortex* and *Persicae Semen*, etc. and is widely used in the treatment of PCOS, infertility, and endometriosis [45]. GFP can increase the pregnancy rate by improving the inflammatory response as well as regulating hormone levels and immune-related protein expression levels to alleviate PCOS-IR, improving ovarian function and promoting ovulation [42, 43]. Zhang found through experiments that the combined use of GFP and ovulation induction drugs for 3 months could significantly improve the ovulation situation and pregnancy rate of PCOS patients [44].

*(3) Clinical Application of Shou Tai Pill and Zi Shen Yu Tai Pill*. Shou Tai Pill (STP) is a classic formula for tonifying the kidneys and calming the fetus, which is taken from the book “Intergrating Chinese and Western Medicine” by the famous physician Zhang Xichun. STP is composed of four herbs: *Corii Asini Colla, Taxilli Herba, Dipsaci Radix, and Cuscutae Semen*, and is widely used in the treatment of abortion, assisted reproduction, and PCOS infertility [45]. Zi Shen Yu Tai Pill (ZYP) is a kind of Chinese medicine preparation based on Shoutai Pill, which is processed by the famous physician and professor Luo Yuankai, according to his personal experience, adding Chinese herbs such as *Polygoni Multiflori Radix, Atractylodis Macrocephalae Rhizoma, and Amomi Fructus*. ZYP is of great significance in assisted reproduction [46]. Due to its remarkable clinical efficacy and high safety without obvious side effects, which was included in the National Basic Medical List in 2018 [47]. ZYP has the function of tonifying the kidney and spleen, nourishing blood and calming fetus, strengthening body and health. Both STP and ZYP have been shown that they have good anti-inflammatory and antioxidant functions, and significantly increase pregnancy and live birth rates in PCOS patients by improving IR, promoting follicle development, and regulating hormone levels [48]. The above studies are listed in Table 3.

#### 2.1.3. Clinical Application of Commonly Used Enema Formulae

Chinese medicine retention enema is a therapeutic means of introducing Chinese medicine with water decoction into the rectum through the anus with a catheter, so that the liquid is absorbed through the colorectum. It avoids the first-pass effect of the liver and increases the drug concentration in the uterus and adnexal areas. Therefore, enema is widely used in clinical infertility caused by PCOS, pelvic inflammatory disease, ovulation disorder, or tubal blockage [49, 50]. A RCT evaluated the efficacy and safety of prepregnancy enemas of Rehabin liquid (a Chinese patent medicine) in combination with mesalazine in women with active ulcerative colitis who had a need for fertility and found that it improved pregnancy outcomes and quality of life [51]. Duan and Lu found that Zichong granules (composed of Rehmanniae Radix Praeparata, Cuscutae Semen, and Chuanxiong Rhizoma, etc.) could increase serum estrogen (E) level and produce estrogen-like effect in mice by rectal administration, thus promoting follicle development [52]. Zhao, C. verified this conclusion that oral administration of Chinese medicine combined with medicine enemas could significantly enhance the ovulation rate in patients with luteinized unrupture follicle syndrome (LUFS) [53].

### 2.2. Mechanism of Chinese Medicine on PCOS Infertility

#### 2.2.1. Improve Hormone Levels and Promote Follicle Development

PCOS patients usually show abnormal sex hormone levels, which can interfere with the normal development of follicles. Liu found that LDP combined with CC could significantly reduce the number of antral follicles, ovarian volume, T, LH, and improve endometrial thickness and E levels in infertile women with PCOS, and enhance pregnancy rate (37.50% vs. 15% *P* < 0.05) [54], which is consistent with Li[55] and Zhang [48]. STP can increase E and progesterone levels, reduce serum D-dimer levels, improve endometrial blood flow, and enhance blastocyst implantation rate to improve the success rate of pregnancy [45, 56]. QUE, the most active compound in ZYP, can improve the aromatase activity of ovarian granulosa cells in high insulin level environment, promote FSH receptor expression, and E synthesis to induce ovulation [57].

#### 2.2.2. Improving IR and Promoting Follicle Development

IR refers to the decrease in the efficiency of insulin uptake and utilization of glucose for various reasons, and the body compensatory secretion of excessive insulin resulting in hyperinsulinemia, to maintain the stability of blood glucose. IR stimulates the production of T in the ovaries and decreases the production of sex hormone binding globulin in the liver, thereby increasing free testosterone (FT) levels in the body [58]. GFP reduces inflammation by altering the structure of the intestinal flora, thereby improving IR [43]. Qiu et al. found that LDP could inhibit PI3/AKT activation, down-regulate mRNA expression of FSHR and Cyp19a1 in ovaries, improve insulin resistance index (HOMA-IR) and sex hormone levels in vivo, alleviate polycystic changes in ovaries, and inhibit premature follicular atresia [39]. Liu et al. came to a conclusion that GFP reduces T, LH and LH/FSH values in PCOS rats by activating the PI3K/AKT/mTOR signaling pathway and inhibiting autophagy of granulation cells, and promoting follicle development and alleviates ovulation disorders in PCOS-IR rats [42]. The network pharmacological analysis showed that ZYP could promote ovulation and improve IR in the treatment of PCOS by inhibiting OS and inflammation response [59].

#### 2.2.3. Improved Pregnancy Outcomes in Ovulation Promotion and Assisted Reproduction

For infertile patients with PCOS who have fertility needs, first-line and second-line treatment such as lifestyle adjustment and ovulation induction therapy are usually preferred. For patients with ineffective treatment, they will eventually choose clinical third-line treatment to assist reproductive technology in order to achieve pregnancy. Chinese medicine significantly improves sex hormone levels, ovulation, and endometrial tolerance in PCOS patients during assisted reproduction, thereby increasing pregnancy and live birth rates. The number of high quality blastocysts significantly increased in 462 patients with expected poor ovarian response (POSEIDON Group 4) undergoing in vitro fertilization-embryo transfer (IVF-ET) after oral administration of Ding kun dan for 5–6 weeks [60]. In a double-blind, multicenter placebo, RCT with a sample size of 2265, administration of ZYP to infertile women undergoing IVF significantly increased the live birth rate compared to placebo before and after ovarian stimulation and ET (26.8% vs. 23.0% (rate ratio [RR], 1.16; 95% CI 1.01–1.34; *P* = .038) [46]. A metabolomic study suggested that ZYP may be involved in the regulation of endometrial proliferation, OS, and lipid metabolism, thus improving endometrial tolerance and oocyte quality and ultimately enhancing IVF live birth rates [140].

### 2.3. Safety of Chinese Medicine in Treating PCOS Infertility

The use of herbal medicines is common in infertile patients with PCOS, while some Chinese herbal medicine (CHM) may contain anthraquinones, flavonoids, and glycosides which are nephrotoxic as well as Polygonum multiflorum Thunb, Tripterygium wilfordii which are hepatotoxic and so on. In addition, they may affect maternal sex hormone levels and may have reproductive toxicity, teratogenic, and abortive adverse effects on the embryo. Most of the experiments did not consider the safety of CHMs. The Chinese medicines involved in this paper, such as BBR, ZYP, and Ding kun Dan, did not show any obvious adverse reactions and some mild adverse reactions such as gastrointestinal tract which occurred in a few patients but could be relieved spontaneously [27, 46, 60]. Therefore, in the treatment of PCOS infertility with TCM, studies on the adverse effects of Chinese medicine and the recommended doses are needed in the future.

## 3. Acupuncture for PCOS with Infertility

Acupuncture is a nondrug therapy in Chinese medicine, which refers to the use of acupuncture or moxibustion to stimulate special parts of the body to regulate the balance of yin and yang in the body and thus achieve the purpose of disease prevention and treatment. This medical method has a mature system and theory, and is based on the internal organs, meridians, qi and blood. As a symbol of TCM, it is being accepted by most countries in the world. Modern research shows that acupuncture cannot directly eliminate disease-causing factors or pathological tissues, but rather prevents or treats disease by activating complex regulatory systems and maintaining physiological homeostasis, thus improving the body's ability to heal itself [61]. The effect on the human body is more likely to promote the robustness of the human body.

In recent years, acupuncture has been increasingly used in the treatment of gynecological diseases due to the advantages of easy and quick operation, better efficacy, and less side effects [62]. Wang, included 27 studies containing 7676 subjects and found that acupuncture was effective in treating infertility, especially in ovulatory disorders and PCOS combined infertility [63]. Quan et al. [64] also concluded that acupuncture or combined with other therapies significantly increased pregnancy and live birth rates in women with PCOS [64]. When acupuncture is applied to gynecological disorders, it can regulate the function of hypothalamic-pituitary-ovarian axis (HPOA), promote ovulation, and improve endometrial tolerance. For summarizing the randomized clinical trials, please refer to Table 4.

### 3.1. Clinical Efficacy of Acupuncture in the Treatment of PCOS with Infertility

Acupuncture includes general acupuncture, electro-acupuncture (EA), warm acupuncture, moxibustion, acupoint injection, and auricular acupuncture. Among them, general acupuncture and EA are the most commonly used treatments in clinical practice, and they are the most effective and widely used; while moxibustion, warm acupuncture and other treatments related to acupuncture are seldom used alone, but mostly in conjunction with acupuncture or herbal supplemental treatments.

#### 3.1.1. General Acupuncture

General acupuncture means inserting filiform needles into special parts of the body (acupoints) at an appropriate angle and using corresponding techniques, such as “lifting and inserting” and “twisting,” to enhance stimulation and achieve better therapeutic effects. Pan et al. used CHM combined with acupuncture as the observation group in an RCT, while the control group was treated with CHM and sham acupuncture. The acupoints mainly included RN4, EX-CA1, and ST29. The results showed that the PR and OR of the observation group were higher than that of the control group (46.34% vs. 18.42%, 58.14% vs. 45.74% *P* < 0.05) [65]. Lai et al. found that CC combined with CHM and acupuncture points RN4, RN3, and EX-CA1 could increase E2 levels, decrease LH and T levels, which can significantly increase PR in PCOS infertile patients compared with CC alone (PR: 46.5% vs. 30.2% *P* < 0.05) [66]. Lai et al. also demonstrated that acupuncture could significantly improve ovulation and normal menstruation rates in PCOS patients compared to controls [67–69]. In infertile patients with PCOS, abnormal hormone levels often result in reduced ER leading to low embryo implantation rate or biochemical pregnancy. Xie, H. also certified in an RCT that the combination of tonifying the kidney, resolving phlegm, and activating blood formula with acupuncture points RN6 and RN4 significantly thickened the endometrial thickness (9.58 ± 0.91 mm vs. 6.43 ± 0.87 mm *P* < 0.05), reduced PI and RI to improve ER and thus to increase PR [70]. He et al. [71] also concluded that acupuncture combined with LET could achieve a complementary and mutually reinforcing effect in significantly improving ER in infertile patients with PCOS [71].

#### 3.1.2. Electroacupuncture

EA is the treatment of stimulating acupuncture points by connecting the needle handle to the electrode after the general acupuncture gets qi and using the EA instrument to output the microcurrent close to the human bio-electricity. The advantage is that the body is stimulated more strongly and consistently, and the acupuncturist is able to objectively control the amount of stimulation the acupuncture provides to the patient. Studies have shown that EA can promote oocyte growth in PCOS patients and increase oocyte maturation and fertilization rates [68, 69]. Li [72] found that EA combined with CHM not only improved the PR in PCOS patients (82.5% vs. 60% *P* < 0.05), but also reduced the incidence of adverse effects such as LUFS and OHSS during ovulatory treatment (2.5% vs. 20%,0% vs. 15.0% *P* < 0.05). Budihastuti et al. [73] draw a similar conclusion that LET combined with EA in ovulation induction therapy for PCOS patients could significantly improve uterine hemodynamics, promote follicle development (19.86 ± 0.7 mm vs. 13.92 ± 3.61 mm *P* < 0.05), and increased endometrial thickness (8.22 ± 1.76 mm vs. 6.95 ± 1.82 mm *P* < 0.05) [73]. In addition, Peng et al. [74] found that EA improved DAEA-induced IR, mitochondrial dysfunction, and endoplasmic reticulum stress in a rat model of PCOS by inhibiting the mTOR/4E-BP1 signaling pathway, and reversed the beneficial effects of EA on PCOS-like rats by inhibiting autophagy in a reversion experiment in which rats with improved symptoms were injected with 3-MA (autophagy inhibitor).

#### 3.1.3. Moxibustion and Warm Acupuncture

According to TCM, deficiency of kidney yang, deficiency of cold in the cellular veins and stasis blocking the thoroughfare and conception vessels are important etiological mechanisms of infertility, and moxibustion is widely used in the improvement of assisted reproduction and metabolic abnormalities in infertile patients with PCOS, because of its effects on warming the menstrual channels and dispersing cold, reinforcing yang and prostration, eliminating stasis and resolving masses, as well as preventing disease and health care. [75] Yu et al. [76] found in an RCT that Chinese medicine combined with moxibustion at points BL23, DU2, DU4 and other acupoints could effectively regulate the level of sexual hormones, improve ovarian hemodynamics, increase OR, and thus improve PR (OR: 84.62% vs. 64.71%, PR: 48.8% vs. 23.53%, *P* < 0.05). Similar to moxibustion, warm acupuncture and moxibustion is a treatment in which moxa wool is twisted around the needle handle and ignited during needle retention, and the needle body transmits heat into the acupoint, which has the effect of warming the meridians and activating qi and blood. In an RCT, Liu et al. [77] found that on the basis of conventional western medicine treatment, the application of warm acupuncture points ST25, RN12 and other acupoints combined with tonifying of kidney and eliminating blood stasis decoction was significantly improved in endometrial thickness (9.85 ± 1.27 mm vs. 7.29 ± 0.93 mm vs. 8.14 ± 1.12 mm *P* < 0.05) and follicle diameter (19.48 ± 2.40 mm vs. 16.36 ± 2.67 mm vs. 17.85 ± 2.28 mm) compared with the two groups of western medicine and CHM, and it improved sex hormone levels, endometrial blood flow parameters, reduced plasma peripheral platelet aggregation rate and D-dimer levels, and increased OR and PRs, and this conclusion is also verified by Xu, et al. [78].

#### 3.1.4. Other Acupuncture-related Therapies

Acupoint injection is a way to treat diseases by injecting drugs into acupoints and organically combining the dual stimulating effects of acupuncture and drugs, which has the characteristics of easy operation, small amount of drugs, and wide indications. Acupoint injection assisted treatment of female infertility can often achieve better therapeutic effects. Cai et al. [79]. found that the injection of urinary gonadotropins into EX-CA1, RN3, RN4 and other acupoint significantly improved the quality and quantity of oocytes compared with intramuscular injection in the buttocks. In addition, PR and OR (PR: 46.7% vs. 26.7, OR: 86.7% vs. 56.7%, *P* < 0.05) could also be significantly improved. Wen et al. verified this experimental conclusion [80]. Auricular acupoint pressing is used to continuously stimulate some specific areas of the auricle by using the seeds of Vaccariae Semen to prevent and treat diseases. Studies have shown that auricular acupoint pressing combined with acupuncture can improve the level of sex hormones in PCOS patients, promote the follicle development and the increase of endometrial thickness, and regulate menstrual cycle [81]. However, there are few trials on acupoint injection, acupoint application, and auricular point, mostly in combination with other therapies, and the level of evidence is not high enough to elucidate the mechanism of action, so future trials with larger sample sizes and more sophisticated designs are needed to prove its effectiveness.

### 3.2. Mechanism of Acupuncture on PCOS Infertility

#### 3.2.1. Regulation of HPOA Function

Modern studies have shown that acupuncture stimulation acts on local skin, the excitation of peripheral nerves is transmitted to the central nervous system, releasing brain neurotransmitters or neuropeptides acting on the HPOA, promoting ovarian vascular dilatation and blood perfusion in ovarian arteries, elevating E and endorphin levels in peripheral blood, and regulating the serum levels of GnRH, LH, FSH, and PRL in PCOS patients [82]. EA improved PCOS-IR by down-regulating hypothalamic NF*κ*B protein expression and significantly reduced abdominal circumference, body weight, serum fasting glucose, fasting insulin, and IR in rats [83]. Zhu et al. [84] found that moxibustion could rescue the HPO axis of ovarian-injured rats, improve hypothalamic GnRH mRNA overexpression and abnormal secretion of reproductive hormones, and maintain normal ovarian function. Huang et al. [85] found that EA could activate the PI3K/AKT signaling pathway, inhibit autophagy-induced follicular atresia, and reduce serum T, LH, and anti-müllerian hormone (AMH) levels, thus improving ovulation. But on the other hand, some studies have also found that acupuncture can inhibit the PI3K/AKT/mTOR pathway by down-regulating LncMEG3 expression, reduce granulocyte autophagy to promote follicle proliferation, and to ensure the normal development of follicles [86]. In TCM theory, acupuncture therapy has a two-way regulation of “activation” and “inhibition” to maintain body homeostasis, so it is assumed that acupuncture treatment also has bidirectional regulation of HPOA function.

#### 3.2.2. Promotion of Ovulation

EA not only promotes angiogenesis in the antral follicles of PCOS rats, but also promotes follicular maturation and ovulation [87]. Yin et al. [88] found that EA combined with ovulatory induction drugs significantly improved the menstrual cycle in infertile patients with PCOS, decreased serum LH, LH/FSH, T and AMH levels, and increased ovulation and PRs. Xiang et al. [89] further found that EA improved oocyte quality and embryonic development potential by activating the IRS-1/PI3K/GLUT4 signaling pathway. Li et al. [90] verified this conclusion that moxibustion improved ovarian function and inhibited ovarian granulosa cell apoptosis by activating the PI3K/AKT signaling pathway. However, this conclusion has also had opposite results. Lai et al. [91] found that EA was able to inhibit the expression of IRS1 and IRS2 mRNA through experiments. In the future, further studies are needed to explore the clinical efficacy and mechanism of acupuncture in PCOS patients.

#### 3.2.3. Improvement of ER

PCOS infertile patients who do not conceive after lifestyle regulation and ovulation induction treatment will eventually opt for ART to achieve pregnancy. However, the cycle PR of assisted reproductive techniques is still hovering at 30%–40% [92]. Owing to sexual hormone disorder and ovulation disorder, PCOS patients often reduce the PR and live birth rate of assisted reproduction due to the low number and poor quality of oocytes obtained, poor ER, and adverse reactions during ovulation induction and embryo transfer. ER refers to the ability of endometrium to accept embryo implantation that changes with menstruation, the influence of sex hormone levels, endometrial thickness, endometrial PI/RI and other factors. EA improves ER by regulating hormone levels and promoting the expression of factors such as vascular endothelial growth factor (VEGF) in the endometrium and ovary, which makes ER present as “trilinear endometrium” and increases pregnancy and embryo transfer rates [93, 94]. Yuan et al. [95] further found that acupuncture combined with CHM can improve the ER of ovulation induction mice by activating the PI3K/Akt/mTOR signaling pathway, down-regulating the expression of miR-494-3p, increasing the expression of endometrial thickness, and ER-related factor HOXA10. Chen et al. [96] validated this conclusion that EA promoted endometrial angiogenesis and thus increased blastocyst implantation rate by activating VEGFR2/PI3K/AKT and VEGFR2/ERK signaling pathways. Shen et al. [97] used high-throughput RNA sequencing and bioinformatics to comparatively analyze patients treated with acupuncture or not and found that circ-SFMBT2, circ-BACH1, and circ-LPAR1 circRNAs were significantly upregulated in patients treated with acupuncture. Therefore, it was also speculated that acupuncture could affect ER by regulating the expression of circRNA, thereby improving the PR and the success rate of ART.

#### 3.2.4. Lose Weight

Studies have shown that 30–70% of PCOS women present with overweight/obesity and visceral obesity, and higher BMI is associated with poorer fertility prognosis [98, 99]. Hypothalamus plays a key role in regulating food intake and energy homeostasis. Studies have shown that the key targets of acupuncture to improve obesity are mainly neurons or neuropeptides in the hypothalamic arcuate nucleus and peripheral hormones (leptin and insulin). Weight loss by reducing leptin and insulin expression to improve leptin and insulin sensitivity [100, 141]. Xu and Zuo [101] found that acupuncture could improve the body mass index (BMI) of infertile patients with PCOS, increase the response of ovulation induction in patients, and effectively shorten the cycle of pregnancy assistance (OR in ovulation promotion cycle: 93.3% vs. 80.0%, clinical PR 43.3% vs. 33.3% *P* < 0.05). Dou et al. [102]. found that acupuncture around the navel increased the PR in obese PCOS patients, which may be related to the increase of serum adiponectin and the decrease of BMI, waist-hip ratio (WHR), homeostasis model of IR, and serum leptin level. In addition, acupuncture can effectively reduce fasting insulin levels and waist-to-hip ratio (WHR) in abdominally obese patients with PCOS, and the therapeutic effect is better than that of diet control plus exercise alone [103]. The theory of TCM believes that acupuncture prescriptions should be “syndrome differentiation and treatment”, and individualized acupuncture prescriptions should be issued according to the actual situation of patients. There may be individual differences in the clinical manifestations of PCOS infertility patients, so individualized acupuncture prescription has a more positive effect on improving pregnancy outcomes in infertile patients with PCOS [104].

#### 3.2.5. Improving Mood

Infertile women with PCOS are often more prone to stress and anxiety due to obesity and infertility, which in turn affect pregnancy outcomes. Therefore, improving patients' mental health and adverse emotions has a significant role in the relief of PCOS symptoms and improvement of pregnancy outcomes. According to the theory of TCM, emotional disorder can easily lead to liver qi stagnation, disorder of liver drainage can lead to deregulation of qi and blood, and disorder of thoroughfare and conception vessels lead to unacceptable pregnancy. Therefore, soothing liver qi and regulating emotions are of great significance for the treatment of infertility. Modern medicine has also clearly demonstrated the effectiveness of psychological interventions in improving anxiety, depression and other adverse emotions, and also can significantly increase the PR [105, 142]. For infertile women with PCOS who are under great social and family pressure, acupuncture has become their best choice due to its less side effects and wide range of action mechanisms, and the combined therapy of acupuncture and Western medicine has been widely used in clinical practice [106]. Wang et al. found that EA improved anxiety/depression symptoms and quality of life in patients with PCOS, and that acupuncture combined with selective 5-hydroxytryptamine (5-HT) inhibitors can significantly improve anxiety compared with selective 5-HT inhibitors alone [107, 108].

### 3.3. The Safety of Acupuncture in Treating PCOS Infertility

Acupuncture therapy is known as a “economic therapy” due to its low invasive, easy to operate, and no gastrointestinal irritation. With the widespread use of acupuncture around the world, there is an increasing trend of reported adverse reactions to acupuncture. Kim et al. [109] believed that adverse infectious events caused by acupuncture may lead to serious consequences, but can be largely avoided if formal acupuncture procedures are followed and aseptic operation is standardized. The safety of acupuncture in the hands of a qualified practitioner is also appreciable. Petra Bäumler's [110] meta-analysis showed that 9.31% (95% CI 5.10% to 14.62%, 11 studies) and 7.57% (95% CI 1.43% to 17.95%, 5 studies) of patients treated with acupuncture had at least one occurrence of a mild acupuncture adverse event, and half of the mild adverse events were pain, bleeding, and ecchymosis at the acupuncture site, while there was no adverse effect on the selection of IVF adjuvant therapy due to infertility. A retrospective cohort study in Korea reported no significant differences in delivery outcomes between the acupuncture and control groups of pregnant women, and the incidence of acupuncture-related adverse events during pregnancy was 1.3%, most of which were mild adverse events such as acupuncture pain [111]. Serious adverse events and fetal complications due to preterm delivery were rare, and the abortion rate was 5%, which was lower than the intervention of the control group [112]. This suggests that acupuncture will not have adverse effects and consequences for pregnant women, so acupuncture can be considered one of the safer medical treatment and can be widely used in patients with PCOS infertility through standardized practice by experienced practitioners.

## 4. Clinical Observation and Mechanism of Other Therapy for PCOS Infertility

In recent years, in addition to the above TCM and acupuncture, the supplement of nutrients and the cultivation of healthy lifestyle, which are also complementary and alternative therapies, have gradually attracted the attention of the researcher. They are impacting the traditional treatment of PCOS infertility and are increasingly accepted and used by infertility patients with PCOS [16].

### 4.1. Clinical Observation and Mechanism of Supplementing Nutrients in the Treatment of PCOS Infertility

Recently, a growing number of studies have found that the lack of vitamins and microelement is related to the occurrence and development of PCOS infertility. Vitamins, minerals, probiotic supplements and other dietary additives can significantly reduce PCOS-related symptoms [113]. The following mainly focuses on the effects of vitamin and microelement supplementation on infertile patients with PCOS.

#### 4.1.1. Vitamin Supplementation

Vitamins are a kind of organic substances that are essential to maintain human life activities, which are mainly involved in the regulation of the body's metabolism. Once deficient, it will cause damage to human health. Currently, vitamin D (VD)and E are the main vitamins that have been studied in the treatment of PCOS infertility.

VD deficiency is very common in women with PCOS. A study by Samantha F Butts et al. found that ovulation and live birth rates were 15.2% and 40% lower, respectively, in VD deficient PCOS patients than in normal women [114]. Recent studies have shown that VD supplementation can increase FSH levels in PCOS patients, thereby decreasing LH/FSH values, and improving IR and hyperlipidemia, thus increasing ovulation and pregnancy rates in PCOS patients [115, 116]. Severe OS is one of the causes of PCOS. Pallavi Dubey et al. found that excessive reactive oxygen species (ROS) in PCOS patients lead to IR, hyperandrogenism (HA), chronic inflammation, and affect oocyte fertilization and blastocyst implantation [113, 117]. Therefore, antioxidative stress may be one of the methods for the treatment of PCOS with infertility. ROS is a natural byproduct of normal oxygen metabolism and plays an important role in cell signaling and homeostasis in the body, and is produced at abnormally high levels in patients with PCOS due to an imbalance between oxidation and antioxidation [118]. VD and vitamin E (VE) can regulate the abnormally high level of ROS in the body. Chen et al. found that short-term VE supplementation can improve OS and reduce ROS levels and reduce the amount of exogenous human menopausal gonadotropin [119]. In addition to the fat-soluble VD and VE, water-soluble vitamins also have antioxidant and anti-inflammatory effects. A study by Szczuko et al. found that water-soluble vitamins could improve the clinical symptoms of PCOS patients by reducing the low-intensity inflammatory response caused by multiple factors such as OS and chronic infection [120].

#### 4.1.2. Microelement Supplementation

Microelements are mineral elements that are present in the body in amounts less than 0.01%. Although their content is minuscule, they play an indispensable role in the human body. Deficiency of a certain microelement inhibits sexual maturity and ovulation function of women, resulting in infertility and threatened abortion. For example, low serum Cu is closely related to recurrent abortion, missed abortion, and spontaneous abortion; low serum Zn can cause a decline in the synthesis and secretion of FSH and LH by the pituitary gland, resulting in ovulation disorders [121]. Relevant studies have found that women can comprehensively play the regulatory function of multiple trace nutrients by supplementing multiple microelements, and jointly play the role of promoting follicle development, preventing multiple birth defects of offspring and improving pregnancy outcomes [122]. Yu et al. found that ovulation and pregnancy rates of infertile patients with PCOS were significantly higher after taking MaFuLong combined with multivitamin tablets compared to the control group taking MaFuLong alone (ovulation rate: 86.67% vs. 63.33%, pregnancy rate: 46.15% vs. 15.79%) [121]. Montanino Oliva et al. also confirmed this conclusion, where women with PCOS showed improvements in their menstrual cycle, ovulation, and body weight after 6 months of continuous administration of a compound with inositol 2 g, L-tyrosine 0.5 mg, folic acid 0.2 mg, selenium 55 mcg, and chromium 40 mcg [123].

### 4.2. The Clinical Efficacy and Mechanism of Healthy Lifestyle in the Treatment of PCOS Infertility

Patients with PCOS are often associated with poor lifestyle habits, and the 2018 edition of the Chinese guidelines for the management of PCOS recommends that the development of a healthy lifestyle (including diet, exercise, and behavioral interventions) should be the preferred treatment for PCOS patients [124].

#### 4.2.1. Lose Weight

The prevalence of obesity in PCOS patients ranges from 30% to 70% and obese PCOS patients are often associated with low fertility and infertility [99, 125]. In addition to unreasonable diet and lack of exercise, PCOS patients are more likely to have abnormal glucose and lipid metabolism due to poor insulin sensitivity, so the probability of obesity is significantly higher than that of normal women [126]. Therefore, a reasonable and efficient weight loss is particularly important for infertile women with PCOS. Weight loss not only improves metabolic abnormalities and insulin sensitivity, but also improves hyperandrogenemia and promotes follicle development. Dietary adjustment is one aspect of the weight loss approach that should not be ignored. Studies have shown that high-carb diet can easily lead to low-level chronic inflammation and obesity in the body, which aggravates ovulation disorders in patients with PCOS infertility [127]. The ketogenic diet is a diet that reduces the proportion of carbohydrate in the diet, and appropriately increases the proportion of vegetables and proteins, which can significantly improve the weight loss effect of PCOS-IR patients in clinical practice. By reducing the absorption of monosaccharide, the ketogenic diet decreases the glucose level in PCOS patients, which in turn lowers insulin levels to regulate glucolipid metabolism and improves the endocrine status [128]. Paoli et al. recruited 24 overweight women with PCOS and after 12 weeks of ketogenic diet, the weight and BMI of the experimental group decreased significantly (pre- and post-treatment weight: 81.19 ± 8.44 kg vs. 71.76 ± 6.66 kg; *p* < 0.0001; pre- and post-treatment BMI: 28.84 ± 2.10 vs. 25.49 ± 1.69; *p* < 0.0001) [129]. A total of 254 PCOS patients with overweight or obesity (BMI ≥25) who were treated with IVF-ET assisted pregnancy by Tan et al. were randomly divided into the weight management strengthening group (80 cases with dietary intervention, the strengthening group), the weight management education group (80 cases, the education group), and the control group (94 cases). After 2 months of treatment, they entered the IVF cycle. BMI, waist circumference, waist-hip ratio, and HOMA-IR of visceral fat area in the strengthening group and the education group were significantly lower than those before weight loss (*P* < 0.05); the trend of the number of oocytes gained, total number of fertilization, total number of cleavage, number of high-quality embryos, and the clinical pregnancy rate among the three groups was strengthening group > education group > control group (*P* < 0.05) [130]. Therefore, weight loss is crucial for infertile patients with PCOS.

#### 4.2.2. Exercise

For different clinical manifestations of PCOS, exercise and weight loss are still the first-line treatment in the world [131]. Li et al. found that aerobic exercise can improve T, E2, and FSH sex hormone disorders in PCOS rats by affecting the hypothalamus-pituitary-ovarian axis, so as to promote follicle development and increase ovulation rate in PCOS patients [132]. Moderate amounts of vigorous exercise are beneficial to most women and may also improve the fertility of infertile patients with PCOS [133]. In aspects of exercise intensity, Cory T. Richards et al. argue that moderate intensity steady state exercise is recommended for PCOS patients compared with high-intensity interval training [134].

In addition to moderate and high intensity exercise, Tai Chi, yoga, Qigong and other exercise methods have also attracted more and more attention from infertile patients with PCOS in recent years. They can not only reduce weight, but also relieve tension and improve immunity of patients. Tai Chi is a traditional Chinese boxing. According to TCM, kidney deficiency is the fundamental pathogenesis of ovulation disorder infertility [135]. Tai Chi focuses on the function of “activating the waist,” “waist is the house of the kidney,” and the exercise of the waist helps the accumulation of the essence of the kidney [136]. Therefore, the practice of Tai Chi helps to cultivate the essence and qi of the kidney, which has a positive effect on infertile patients with PCOS from the perspective of TCM. Yoga and Tai Chi are very similar limbs and trunk exercise therapies in the two ancient medical systems of China and India. Studies have found that infertile patients with PCOS are more prone to anxiety and depression than those with tubal factor infertility [137]. In a RCT conducted by Maryam Mohseni et al., they found that yoga practice could significantly reduce hypertrichosis and WHR in PCOS patients, and it was recommended to include it in the treatment strategy of PCOS women. In addition to Tai Chi and yoga, Qigong, as a unique Chinese gymnastics with health care, wellness, and disease elimination effects, is now widely used in disease prevention, treatment, and rehabilitation. Qigong not only promotes the circulation of qi and blood, but also regulates the emotions and relieves negative emotions through exercise. Sun Xiaoling et al. found that exercise can reduce IR, promote the recovery of ovarian function, and increase ovulation rate in PCOS patients, thus improving pregnancy rate [139]. Therefore, regular and moderate exercise is very important for infertile patients with PCOS.

## 5. Conclusion

This review mainly discusses the therapeutic effects of CHMs, acupuncture, nutrient supplementation, healthy lifestyle, Tai Chi, yoga, and Qigong of CAM on infertile women with PCOS. The results showed that CAM can improve IR and sex hormone disorders, enhance endometrial thickness, increase ovulation rate, pregnancy rate, and improve anxiety status in infertile patients with PCOS. At present, few studies have reported the adverse effects of CAM on liver and kidney function and fertility outcomes of infertile women with PCOS, and few reports of abortion and malformation of embryos. CAM can not only regulate the current physical and mental health status of infertile patients with PCOS, but also regulate many long-term complications caused by PCOS. Overall, CAM is a safe treatment option for infertile patients with PCOS. However, due to the limited number of methods and trials included and the generally low quality, CAM needs to be studied in more depth and larger trials to demonstrate its efficacy and safety if it is to be used more widely in the clinic and mostly as an auxiliary to primary therapy for therapeutic purposes.

## Figures and Tables

**Table 1:** List of reference numbers corresponding to traditional Chinese medicine, acupuncture and other therapies.

CAM treatment modalities	Mechanism of action	Reference range
CHM (monomers, compound, compound enema)	Monomers (BBR, CRY, QUE), compound (BSD, GFP, STP, and ZYP) in the treatment of patients with PCOS infertility is to improve IR and glucose and lipid metabolism disorder, improve sex hormone levels, and promote follicular development	[17–60]
Acupuncture (general acupuncture, EA, moxibustion and warm acupuncture, other treatment modalities related to acupuncture)	Lose weight, improves depression, regulates the HPOA, promotes ovulation, and improves ER.	[61–112]
Other therapies (vitamin and trace element supplementation and other nutrients, weight loss, exercise, and other healthy lifestyles)	Nutrient supplementation improves FSH levels and chronic low-intensity inflammatory response, and micronutrient supplementation promotes paired follicle development in PCOS infertility patients. Weight loss can improve insulin sensitivity, HA, and follicular development in PCOS infertility patients. Exercise modulates the HPOA to improve sex hormone disorders and increase ovulation and pregnancy rates in patients with PCOS infertility.	[113–139]

**Table 2:** RCT and results of monomers of CHM.

ID	Design	Test subjects	Sample size	Grouping situation	Outcomes	Ethical clearance number
24	RCT	Human	78	Control group: Non-PCOS infertility	Observation group B: mTOR mRNA ↓ (*P* = 0.001), IRS-1 mRNA ↑ (*P* = 0.009)	[2017] No. 158
				Observation group A : PCOS infertility		
				Observation group B: PCOS infertility + BBR		
27	RCT	Human	644	Berberine group(A): BBR + LET placebo	The live birth rate, PR and OR of B and C were similar and significantly higher than those of berberine group.	ChiCTR-TRC-09000376
				LET group(B): LET + BBR placebo		
				Combination group(C): LET + BBR		
34	RCT	Human	84	Treatment group: QUE	Treatment group: adiponectin ↑ HOMA-IR ↓、 T ↓、 LH ↓、 FBS ↓ All <0.009	IRCT2013112515536N1
				Control group: Placebo		
35	RCT	Human	78	Treatment group: QUE	Resistin concentration: 2.07 ± 0.23 vs. 2.88 ± 0.40 ng/ml mRNA levels: 0.64 ± 0.58 vs. 1 ± 0.56	IRCT2016082215536N4
				Control group: Placebo	T: 0.72 ± 0.15 vs. 0.76 ± 0.12 ng/ml LH: 8.05 ± 2.88 vs. 8.77 ± 1.99 mIU/ml	
					All *p* < 0.05	
31	RCT	Rats	60	Normal rats:	Observation group B: T↓、E2↓、 LH↓、 LH/FSH ↓ Inhibin B、 lutein mRNA and protein expression ↓ Activin A mRNA and protein expression ↑	All protocols were conducted in accordance with the guidance suggestions for the care and use of laboratory animals, formulated by the Ministry of Science and Technology of China.
				PCOS rat A: Normal saline		
				PCOS rat B: CRY		
					All *P* < 0.05	
32	RCT	Rats	60	Blank control group	Weight ↓, ovarian mass ↓, LH ↓, LH/FSH ↓, T ↓, TNF-*α* ↓	No. 20190103
				PCOS group		
				PCOS + HMGB1 group		
				PCOS + HMGB1 + CRY		
36	RCT	Rats	24	Control group (C): carboxymethyl cellulose aqueous solution	T↓ : *Q* + *L*:0.78 ng/ml ± 0.14 vs. M:1.69 ng/ml ± 0.17	BAS#0256
				PCOS group (M): LET	E2↓ : *Q* + *L*:8.85 pg/ml ± 0.19 vs. M:1.61 pg/ml ± 0.29)	
				Metformin group (*M* + *L*): metformin + LET quercetin group (*Q* + *L*): QUE + LET	P↑ : *Q* + *L*:34.47 ng/ml ± 1.65 vs. M:1.08 ng/ml ± 1.17	
					All *P* < 0.05	

**Table 3:** RCT and results of TCM compound.

ID	Design	Model	Sample size	Grouping situation	Outcome	Ethical clearance number
54	RCT	Human	80	Observation group: Daing 35 + CC Control group: Daying 35 + LDP	PR: 37.50% (15/40) vs. 15% (6/40) Number of follicles ↓、ovarian volume ↓、T ↓、 LH ↓ Endometrial thickness ↓、E2↑ *P* < 0.05	This trial was approved by the Ethics Committee of Zijin county maternal and Child Health hospital, Guangdong Province, and the ethical approval number is not explicitly mentioned in the text
44	RCT	Human	56	Observation group: metformin + ethinyl estradiol cyproterone tablets + GFP Control group: Metformin + ethinyl estradiol cyproterone tablets	Total efficiency: 96.4% (27/28) vs. 71.4% (20/28) pregnancy rate: 67.9% (19/28) vs. 35.6% (10/28) All *P* < 0.05	This trial was approved by the ethics committee of Anyang People's hospital in Henan Province; the ethics approval number is not explicitly mentioned in the text
55	RCT	Human	100	Observation group: ethinyl estradiol cyproterone tablets + ZYP Control group: ethinylestradiol cyproterone tablets	Total efficiency: 100% (50/50) vs. 80% (40/50) LH/FSH<3 : 100%- (50/50) vs. 80% (40/50) All *P* < 0.05	This trial was approved by the Ethics Committee of the Dazhou hospital of integrative medicine, the ethical approval number is not explicitly mentioned in the text
48	RCT	Human	64	Observation group: estradiol valerate tablets + progesterone capsules + ZYP Control group: estradiol valerate tablets + progesterone capsules	Pregnancy rate: 40.63% (13/32) vs. 15.63%(5/32) survival rate: 28.13% (9/32) vs. 3.13% (1/32) All *P* < 0.05	This trial was approved by the thics Committee of Nantong Chinese hospital, the ethical approval number is not explicitly mentioned in the text
49	RCT	Human	60	Observation group: Chinese herbal enema (*CyperusrotundusL, Lindera aggregata (Sims) Kosterm, Amomum villosum Lour, Radix Aucklandiae, Olibanum, Geosaurus, Bombyx Batryticatus, Curcuma phaeocaulis Valeton, Angelica sinensis (Oliv.) Diels, Salvia miltiorrhiza Bunge.)* Blank control group (K)	Uterine spiral artery RI: 0.63 ± 0.03 vs. 0.66 ± 0.03 S/D: 2.72 ± 0.17 vs. 3.06 ± 0.22) Ovarian spiral artery RI: 0.60 ± 0.04 vs. 0.56 ± 0.04 S/D: 2.47 ± 0.3 vs. 2.28 ± 0.08	This trial has been approved by the Ethics Committee of Suzhou hospital of traditional Chinese medicine, Jiangsu Province, and the ethics approval number is not explicitly mentioned in the text
39	RCT	Rats	50	Blank control group (K) High fat diet (HFD) + LET HFD + LET + metformin Low-dose group: HFD + LET + LDP High-dose group: HFD + LET + LDP	In PCOS-IR rats with upregulated ovarian FSHR and Cyp19a1 mRNA levels, LDP (3.6 g-kg-1-d-1) significantly reversed the upregulated phosphorylation of IRS-1 (S307) and the downregulated phosphorylation of PI3Kp85*α*, Akt and FoxO1a.	This trial has been approved by the ethics committee of the iangsu key laboratory for the evaluation and translation of traditional Chinese medicine, and the ethics approval number is not explicitly mentioned in the text
43	RCT	Rats	72	Blank control group (K) Model control group (M) GFP low dose group (D) GFP medium dose Group (Z) GFP high dose group (Group G) Positive drug (metformin) control group (group Y)	Experimental group HS-CPR ↓, IL-6 ↓, TNF-*α* ↓, ucg-008 ↑, nk4a136 ↑	SYXK2018-0126
42	RCT	Rats	84	Control group PCOS model group(M) low-dose GFP group.(D) The medium-dose GFP group.(Z) High-dose GFP group(G) Metformin group(group Y). Medium dose GFP plus LY294002 group	GFP treatment group: atresia follicles ↓, cystic follicles ↓, mature follicles ↑ and corpus luteum ↑ T ↓、 LH ↓、 FINS ↓、 LH/FSH↓、HOMA-IR ↓ Phosphorylation levels of PI3K, AKT and mTOR↑ All *P* < 0.05	SYXK2018-0126

Blank control group (K), model control group (M), low dose group (D), medium dose group (Z), high dose group (G), positive drug (Y).

**Table 4:** RCT and results of acupuncture.

ID	Design	Sample size	Interventions	Outcomes	Composition	Ethical clearance
67	RCT	86	Manual acupuncture: CHM + acupuncture sham acupuncture: CHM	PR:46.34% (19/41) vs. 18.42% (7/38) *P* < 0.05	Acupoints: RN4, EX-CA1, ST29, ST36, SP6 Prescription: not mentioned	2017-569-52–01
68	RCT	86	Observation group: CC + CHM + acupuncture Control group: CC treatment	PR: 46.5% (20/43) vs. 30.2% (13/43) Early miscarriage rate: 15.0% (3/20) vs. 38.5% (5/13) Observation group: E2↑, T↓, LH↓ All *P* < 0.05	Prescription: *Medicata Fermentata Massa, Citri Reticulatae Pericarpium, Aurantii Fructus, Cyperi Rhizoma, Atractylodis Rhizoma, Pinelliae Rhizoma, Zingiberis Recens Rhizoma, Glycyrrhizae Radix et Rhizoma, Poria, Arisaema cum Bile* Acupoints: RN4, RN3, EX-CA1, ST29, SP6, ST36, SP10	This trial was approved by the Ethics Committee of The First People's hospital of Foshan city, Guangdong Province, and the ethical approval number is not explicitly mentioned in the text
69	RCT	60	Observation group: LET + acupuncture Control group: LET	PR: 60.53% (23/38) vs. 27.03% (10/37) OR: 89.47% (34/38) vs. 67.57% (25/37) All *P* < 0.05	Acupoints: EX-CA1, SP6, RN3	This trial was approved by the Ethics Committee of Affiliated hospital of Shandong University of traditional Chinese medicine, and the ethical approval number is not explicitly mentioned in the text
72	RCT	60	Observation group: Daying 35 + LET + CHM Control group: Daing 35 + LET	Endometrial thickness: 9.58 ± 0.91 mm vs. 6.43 ± 0.87 mm PR: 36.67% (11/30) vs. 23.33% (7/30) observation group: RI↓, PI↓ All *P* < 0.05	Prescription: *Angelicae Sinensis Radix, Paeoniae Alba Radix, Rehmanniae Radix Praeparata, Dioscoreae Rhizoma, Cuscutae Semen, Dipsaci Radix, Epimedii Herba, Cremastrae seu, Pleiones, Pseudobulbus, Gleditsiae Spina, Salviae Miltiorrhizae, Radix et Rhizoma, Spatholobi Caulis* Acupoints: RN4, RN3, EX-CA1, ST28, KI3, ST40, SP6 Moxibustion points: RN4, RN3, EX-CA1, ST28.	Trial was approved by Ganzhou traditional Chinese medicine hospital and the ethical approval number is not explicitly mentioned in the text
73	RCT	120	A: Daphne + LET + left right return pill + EA group B: Daphne + left and right return pill + EA C: Daphne + LET + EA	Clinical efficacy: 85.0% (43/40) vs. 70.0% (28/40) vs. 60.0% (24/40) Type a endometrium: 65.0% (26/40) vs. 35.0% (14/40) vs. 35% (14/40) OR: 40.0% (16/40) vs. 30.0% (12/40) vs. 20.0% (8/40) PR: 22.5% (9/40) vs. 12.5% (5/40) vs. 10.0% (4/40) All *P* < 0.05	Acupoints: RN4, RN3, RN6, EX-CA1, SP10, ST36, SP6, KI3, KI6	Trial was approved by Southwest medical university affiliated traditional Chinese medicine hospital and the ethical approval number is not explicitly mentioned in the text
80	RCT	103	Observation group: CC + CHM + moxibustion Control group: CC	Observation group: The peak systolic flow rate↑, PI↑ RI ↓ OR: 84.62% (44/52) vs. 64.71% (33/51) PR: 48.8% (25/52) vs. 23.53% (12/51) All *P* < 0.05	Prescription: *Angelicae Sinensis Radix, Paeoniae Alba Radix, Rehmanniae Radix, Praeparata, Corni Fructus, Ligustri Lucidi Fructus, Testudinis, Carapax et Plastrum, Ecliptae Herba, Glycyrrhizae, Radix et Rhizoma* Moxibustion: DU2, BL23, DU4	Trial was approved by Department of Gynecology, directly under the authority No.2 outpatient department, Henan Province and the ethical approval number is not explicitly mentioned in the text
81	RCT	90	Observation group: Aspirin + CC + CHM + warm acupuncture Control group A: Aspirin + CC Control group B: Aspirin + CC + CHM	Endometrial thickness: 9.85 ± 1.27 mm vs. 7.29 ± 0.931 mm vs. 8.14 ± 1.12 mm Follicle diameter: 19.48 ± 2.40 mm vs. 16.36 ± 2.67 mm vs. 17.85 ± 2.28 mm OR: 90.0% (27/30) vs. 63.3% (19/30) vs. 70.0% (21/30) PR:46.7% vs. 16.7% (5/30)vs. 20.0% (6/30) All *P* < 0.05	Prescription: *Dioscoreae Rhizoma, Rehmanniae Radix, Praeparata, Lycii Fructus, Corni Fructus, Cuscutae Semen, Morindae Officinalis, Radix, Epimedii Herba, Hominis Placenta, Spatholobi Caulis, Salviae Miltiorrhizae Radix et Rhizoma, Dipsaci Radix,* Acupoints: ST25, RN12, RN4, RN3, RN6, SP10, EX-CA1, LR3, SP6, ST36	Trial was approved by Tangshan traditional Chinese medicine hospital and the ethical approval number is not explicitly mentioned in the text
82	RCT	82	Observation group: CC + Duoyuan acupuncture Control group: CC	PR: 51.2% (21/41) vs. 26.8% (11/41) Bilateral ovarian volume↓, follicle number↓, menstrual cycle↓, All *P* < 0.05	Acupoints: RN12, RN4, RN6, RN3, DU4, DU3, DU2	Trial was approved by Nanjing University of traditional Chinese medicine and the ethical approval number is not explicitly mentioned in the text
83	RCT	60	Observation group: Acupoint injection of urotropin injection Control group: Urinary gonadotropin injection into the gluteal muscle.	FSH: 11.36 ± 1.84 IU/L vs. 9.87 ± 1.75 IU/L T: 0.72 ± 0.1 *μ*g/L vs. 1.18 ± 0.16 *μ*g/L LH: 19.36 IU/L ± 4.25 vs. 24.18 ± 4.16 IU/L All *P* < 0.05	Acupoints: EX-CA1, RN3, RN4, SP6	Trial was approved by Chenghai district People's hospital, Shantou City, Guangdong Province and the ethical approval number is not explicitly mentioned in the text
84	RCT	80	Observation group: CC + Chinese medicine + acupuncture point injection of angelica injection Control group: CC	Total efficiency: 80.0% (32/40) vs. 52.5% (21/40) Endometrial thickness: 0.98 ± 0.06 cm vs. 0.74 ± 0.05 cm All *P* < 0.05 After treatment, there was a statistically significant difference in the TCM symptom scores between the treatment and control groups	Chinese herbs: *Cuscutae Semen, Morindae Officinalis Radix, Cistanches Herba, Cinnamomi Ramulus, Astragali Radix, Liquidambaris Fructus, Cudraniae Radix, Salviae Miltiorrhizae, Radix et Rhizoma, Cyperi Rhizoma, Curcumae Rhizoma, Arecae Pericarpium* Acupuncture point injection: BL23	Trial was approved by Panyu district central hospital, Guangzhou city, guangdong province and the ethical approval number is not explicitly mentioned in the text
85	RCT	125	Observation group: estradiol valerate tablets + acupuncture + ear acupuncture Control group: estradiol valerate tablets	Total efficiency: 93.65% (59/63) vs. 80.65% (50/62) Time to return to normal menstruation: 3.71 ± 0.84 months vs. 4.29 ± 1.06 months OR: 90.46% (57/63) vs. 77.42% (48/62) All *P* < 0.05	Acupoints: RN6, SP6, RN4, BL20, ST36, LR3, DU4, BL23 Ear points: liver, kidney, ovary, ovaries and endocrine, with matching points for spleen, lumbar, and pelvic	Trial was approved by Sichuan Nanchong traditional Chinese medicine hospital and the ethical approval number is not explicitly mentioned in the text
92	RCT	120	C: Daing 35 + acupuncture B: Daying 35 + CHM A : Daing 35	Total efficiency: 95.0% (39/40) vs. 85.0% (34/40) vs. 72.5% (29/40) PR: 80.0% (32/40) vs. 62.5% (25/40) vs. 52.5% (21/40) All *P* < 0.05	Prescription: *Epimedii Herba, Cuscutae Semen, Fluoritum, Atractylodis Rhizoma, Citri Reticulatae, Pericarpium, Pinelliae Praeparatum, Rhizoma, Coicis Semen, Poria, Angelicae Sinensis Radix, Paeoniae Alba Radix, Chuanxiong Rhizoma, Cyperi, Rhizoma, Aurantii, Fructus* Acupoints: RN6, RN4, EX-CA1, ST25, ST36, SP6, SP9, SP10, ST40	Trial was approved by Hubei maternal and child health hospital traditional Chinese medicine and the ethical approval number is not explicitly mentioned in the text
105	RCT	60	Observation group: Daying 35 + acupuncture Control group: Daing 35	OR: 93.3% (28/30) vs. 80.0% (24/30) Clinical PR 43.3% (13/30) vs. 33.3% (10/30) Observation group: E2↓、T↓、BMI↓ All *P* < 0.05	Acupoints: RN4, RN6, SP6, ST36, EX-CA1, BL23, BL20, BL21, BL18	Trial was approved by Lianyungang maternal and child health hospital reproductive medicine center and the ethical approval number is not explicitly mentioned in the text
106	RCT	96	Observation group: acupuncture Control group: LET	Observation group: PR↑, APN↑, BMI↓, WHR↓, HOMA-IR↓, LEP↓ All *P* < 0.05	Acupoints: RN9, RN7, ST25, ST26, ST24	Trial was approved by reproductive center of the first affiliated hospital of Tianjin University of traditional Chinese medicine and the ethical approval number is not explicitly mentioned in the text
93	RCT	76	Observation group: EA Control group: Sham acupuncture	Transferable embryo rate: 49.0% (284/580) vs. 41.9% (273/652) High quality embryo rate: 36.6% (104/284) vs. 27.8% (76/273) Live birth rate: 50% (19/38) vs. 26.3% (10/38) Phlegm-damp syndrome score↓, IR↓, IRS-1↑, PI3K↑, GLUT4 mRNA↑ All *P* < 0.05	Acupoints: RN12, ST25, SP15, GB26, RN6, RN4, SP10, ST40, ST36, SP9	SDSZYYSZ20170210
107.	RCT	60	Observation group: dietary control plus exercise + acupuncture Control group: dietary control plus exercise	Observation group: Fasting insulin↓, fasting glucose↓ and waist↓ *P* < 0.05	Acupoints: GB26, ST25, SP15, BL23, BL32, ST29, GB41, SJ5	no.2018019
76	RCT	80	Observation group: Daying 35 + CHM Control group: Daing 35	Fertility rate: 82.5% (33/40) vs. 60% (24/40) LUFS: 2.5% (1/40) vs. 20% (8/40) OHSS: 0% (0/40) vs. 15.0% (6/40) All *P* < 0.05	Prescription: *Atractylodis Rhizoma, Epimedii Herba, Cuscutae Semen, Paeoniae Alba Radix, Coicis Semen, Pinelliae Praeparatum, Rhizoma* *, Angelicae Sinensis, Radix, Fluoritum, Cyperi Rhizoma, Chuanxiong Rhizoma, Poria, Citri Reticulatae Pericarpium* Acupoints: RN17, SP6, LR3, ST25, ST36, EX-CA1, RN12, RN4, LR14	Trial was approved by Zhaoqing Gaoyao People's hospital and the ethical approval number is not explicitly mentioned in the text

## Data Availability

All data included in this study are available upon request by contact with the corresponding author.

## Abbreviations

PCOS: Polycystic ovary syndrome
IR: Insulin resistance
HOMA-IR: Insulin resistance index
VE: Vitamin E
OS: Oxidative stress
FSH: Follicle stimulating hormone
LH: Luteinizing hormone
ART: Assisted reproductive technology
VD: Vitamin D
5-HT: 5-hydroxytryptamine
CAM: Complementary and alternative medicine
HPOA: Hypothalamic-pituitary-ovarian axis
TCM: Traditional Chinese medicine
CHM: Chinese herbal medicine
BBR: Berberine
WHR: Waist-to-hip ratio
CC: Clomiphene
LET: Letrozole
CRY: Cryptotanshinone
PR: Pregnancy rate
ASD: Androstenedione
T: Testosterone
QUE: Quercetin
LDP: Liu Wei Di Huang Prescription
GFP: Gui Zhi Fu Ling Pill
PCOS-IR: Polycystic ovary syndrome with insulin resistance
STP: Shou Tai Pill
ZYP: Zi Shen Yu Tai Pill
LUFS: Luteinized unruptured follicle syndrome
E: Estrogen
CPA: Cyproterone acetate
OR: Ovulation rate
IVF-ET: In vitro fertilization-embryo transfer
RCT: Randomized controlled trial
PI: Perfusion index
RI: Resistive index
ER: Endometrial receptivity
EA: Electroacupuncture
OHSS: Ovarian hyperstimulation syndrome
GnRH: Gonadotropin-releasing hormone
AMH: Anti-müllerian hormone
VEGF: Vascular endothelial growth factor
BMI: Body mass index
ROS: Reactive oxygen species.

## References

[1] Walter K. (2022). What is polycystic ovary syndrome?. *JAMA*.

[2] Stener-Victorin E., Deng Q. (2021). Epigenetic inheritance of polycystic ovary syndrome—challenges and opportunities for treatment. *Nature Reviews Endocrinology*.

[3] Garg A., Patel B., Abbara A., Dhillo W. S. (2022). Treatments targeting neuroendocrine dysfunction in polycystic ovary syndrome (PCOS). *Clinical Endocrinology*.

[4] Joham A. E., Teede H. J., Ranasinha S., Zoungas S., Boyle J. (2015). Prevalence of infertility and use of fertility treatment in women with polycystic ovary syndrome: data from a large community-based cohort study. *Journal of Women’s Health*.

[5] Vander Borght M., Wyns C. (2018). Fertility and infertility: definition and epidemiology. *Clinical Biochemistry*.

[6] Li Y., Ruan X., Wang H. (2018). Comparing the risk of adverse pregnancy outcomes of Chinese patients with polycystic ovary syndrome with and without antiandrogenic pretreatment. *Fertility and Sterility*.

[7] Costello M. F., Misso M. L., Balen A. (2019). A brief update on the evidence supporting the treatment of infertility in polycystic ovary syndrome. *The Australian and New Zealand Journal of Obstetrics and Gynaecology*.

[8] Sadeghi H. M., Adeli I., Calina D. (2022). Polycystic ovary syndrome: a comprehensive review of pathogenesis, management, and drug repurposing. *International Journal of Molecular Sciences*.

[9] Popovic M., Sartorius G., Christ-Crain M. (2019). Chronic low-grade inflammation in polycystic ovary syndrome: is there a (patho)-physiological role for interleukin-1?. *Seminars in Immunopathology*.

[10] Wode K., Henriksson R., Sharp L., Stoltenberg A., Hok Nordberg J. (2019). Cancer patients’ use of complementary and alternative medicine in Sweden: a cross-sectional study. *BMC Complementary and Alternative Medicine*.

[11] Berna F., Goritz A. S., Mengin A., Evrard R., Kopferschmitt J., Moritz S. (2019). Alternative or complementary attitudes toward alternative and complementary medicines. *BMC Complementary and Alternative Medicine*.

[12] James P. B., Taidy-Leigh L., Jawo Bah A., Sam Kanu J., Bainga Kangbai J., Sevalie S. (2018). Prevalence and correlates of herbal medicine use among women seeking Care for Infertility in Freetown, Sierra Leone. *Evidence-Based Complementary and Alternative Medicine*.

[13] Dehghan M., Mokhtarabadi S., Heidari F. G. (2018). Complementary and alternative medicine usage and its determinant factors among Iranian infertile couples. *Journal of Complementary and Integrative Medicine*.

[14] Özkan F. S., Karaca A., Sarak K. (2018). Complementary and alternative medicine used by infertile women in Turkey. *African Journal of Reproductive Health*.

[15] Hwang J. H., Kim Y. Y., Im H. B., Han D. (2019). Complementary and alternative medicine use among infertile women attending infertility specialty clinics in South Korea: does perceived severity matter?. *BMC Complementary and Alternative Medicine*.

[16] Feng J., Wang J., Zhang Y. (2021). The efficacy of complementary and alternative medicine in the treatment of female infertility. *Evidence-based Complementary and Alternative Medicine*.

[17] Sönmez S., Ozturk M., Sonmez F. (2021). Prevalence and predictors of the usage of complementary alternative medicine among infertile patients. *Journal of Gynecology Obstetrics and Human Reproduction*.

[18] Yu J., Yu C., Cao Q. (2017). Consensus on the integrated traditional Chinese and Western medicine criteria of diagnostic classification in polycystic ovary syndrome (draft). *Journal of Integrative Medicine*.

[19] Zhou K., Zhang J., Xu L., Wu T., Lim C. E. D. (2016). Chinese herbal medicine for subfertile women with polycystic ovarian syndrome. *Cochrane Database of Systematic Reviews*.

[20] Luo Q., Tan Y., Hu R., Xia Y., Xia G. (2021). Mechanism of *Ziyin* recipe for treatment of ovulatory infertility: a network pharmacology-based study and clinical observations. *Journal of Southern Medical University*.

[21] Bai Y. l., Chen Y., Jiang C. (2021). Efficacy and safety of traditional Chinese medicine in the treatment of immune infertility based on the theory of “kidney deficiency and blood stasis”: a systematic review and meta-analysis. *Evidence-based Complementary and Alternative Medicine*.

[22] Moini Jazani A., Nasimi Doost Azgomi H., Nasimi Doost Azgomi A., Nasimi Doost Azgomi R. (2019). A comprehensive review of clinical studies with herbal medicine on polycystic ovary syndrome (PCOS). *Daru Journal of Pharmaceutical Sciences*.

[23] Kumar A., Ekavali, Chopra K., Mukherjee M., Pottabathini R., Dhull D. K. (2015). Current knowledge and pharmacological profile of berberine: an update. *European Journal of Pharmacology*.

[24] Kuang H., Duan Y., Li D. (2020). The role of serum inflammatory cytokines and berberine in the insulin signaling pathway among women with polycystic ovary syndrome. *PLoS One*.

[25] Zhang S. w., Zhou J., Gober H. J., Leung W. T., Wang L. (2021). Effect and mechanism of berberine against polycystic ovary syndrome. *Biomedicine & Pharmacotherapy*.

[26] Ming J., Yu X., Xu X. (2021). Effectiveness and safety of Bifidobacterium and berberine in human hyperglycemia and their regulatory effect on the gut microbiota: a multi-center, double-blind, randomized, parallel-controlled study. *Genome Medicine*.

[27] Wu X. K., Wang Y. Y., Liu J. P. (2016). Randomized controlled trial of letrozole, berberine, or a combination for infertility in the polycystic ovary syndrome. *Fertility and Sterility*.

[28] Wang C. (2019). Cryptotanshinone alleviates OVA-induced airway inflammation in asthmatic mice via TWEAK/Fn14 and TGF-*β*1/Smads signaling pathways. *Chinese Pharmacological Bulletin*.

[29] Kuang H., Ma K., Li W. (2017). The mechanism of cryptotanshinone regulating the reproductive endocrine function of ovarian granulosa cells in PCOS model rats. *Chinese Journal of Medicine*.

[30] Ong M., Peng J., Jin X., Qu X. (2017). Chinese herbal medicine for the optimal management of polycystic ovary syndrome. *The American Journal of Chinese Medicine*.

[31] Xia Y., Zhao P., Huang H., Xie Y., Lu R., Dong L. (2017). Cryptotanshinone reverses reproductive disturbances in rats with dehydroepiandrosterone-induced polycystic ovary syndrome. *American Journal of Tourism Research*.

[32] Yang Y., Yang L., Qi C. (2020). Cryptotanshinone alleviates polycystic ovary syndrome in rats by regulating the HMGB1/TLR4/NF-*κ*B signaling pathway. *Molecular Medicine Reports*.

[33] Pourteymour Fard Tabrizi F., Hajizadeh-Sharafabad F., Vaezi M., Jafari-Vayghan H., Alizadeh M., Maleki V. (2020). Quercetin and polycystic ovary syndrome, current evidence and future directions: a systematic review. *Journal of Ovarian Research*.

[34] Rezvan N., Moini A., Janani L. (2016). Effects of quercetin on adiponectin-mediated insulin sensitivity in polycystic ovary syndrome: a randomized placebo-controlleddouble-blind clinical trial. *Hormone and Metabolic Research*.

[35] Khorshidi M., Moini A., Alipoor E. (2018). The effects of quercetin supplementation on metabolic and hormonal parameters as well as plasma concentration and gene expression of resistin in overweight or obese women with polycystic ovary syndrome. *Phytotherapy Research*.

[36] Jahan S., Abid A., Khalid S. (2018). Therapeutic potentials of Quercetin in management of polycystic ovarian syndrome using Letrozole induced rat model: a histological and a biochemical study. *Journal of Ovarian Research*.

[37] Chen W., Wang J., Shi J. (2019). Longevity effect of Liuwei Dihuang in both *Caenorhabditis elegans* and aged mice. *Aging and disease*.

[38] Sun X., Wu B., Geng L., Zhang J., Qin G. (2021). Xiaokang Liuwei Dihuang decoction ameliorates the immune infertility of male rats induced by lipopolysaccharide through regulating the levels of sex hormones, reactive oxygen species, pro-apoptotic and immune factors. *Biomedicine & Pharmacotherapy*.

[39] Qiu Z., Dong J., Xue C. (2020). Liuwei Dihuang Pills alleviate the polycystic ovary syndrome with improved insulin sensitivity through PI3K/Akt signaling pathway. *Journal of Ethnopharmacology*.

[40] Zeng J., Zhang X., Wang J., Cheng X., Zhang Y., Zhou W. (2020). Comparison of donepezil, memantine, melatonin, and liuwei dihuang decoction on behavioral and immune endocrine responses of aged senescence-accelerated mouse resistant 1 mice. *Frontiers in Pharmacology*.

[41] He D., Huang J., Zhang Z. (2019). A network pharmacology-based strategy for predicting active ingredients and potential targets of LiuWei DiHuang pill in treating type 2 diabetes mellitus. *Drug Design, Development and Therapy*.

[42] Liu M., Zhu H., Zhu Y., Hu X. (2021). Guizhi Fuling Wan reduces autophagy of granulosa cell in rats with polycystic ovary syndrome via restoring the PI3K/AKT/mTOR signaling pathway. *Journal of Ethnopharmacology*.

[43] Zhu Y., Li Y., Liu M., Hu X., Zhu H. (2020). Guizhi Fuling Wan, Chinese herbal medicine, ameliorates insulin sensitivity in PCOS model rats with insulin resistance via remodeling intestinal homeostasis. *Frontiers in Endocrinology*.

[44] Zhang Y. (2019). Guizhi fuling capsules combined with ethinylestradiol cyproterone tablets and metformin in the treatment of 56 cases of polycystic ovary syndrome. *Northern Pharmacy*.

[45] Li H. F., Shen Q., Li X. (2020). The Efficacy of Traditional Chinese Medicine Shoutai Pill combined with Western Medicine in the first trimester of pregnancy in women with unexplained recurrent spontaneous abortion: a systematic review and meta-analysis. *BioMed Research International*.

[46] Chen X., Hao C., Deng W. (2022). Effects of the Zishen Yutai pill compared with placebo on live births among women in a fresh embryo transfer cycle: a randomized controlled trial. *Obstetrics & Gynecology*.

[47] Zuo W., Mei D., Sun W. (2020). The interpretation of China national essential medicines list 2018. *Expert Review of Clinical Pharmacology*.

[48] Zhang L. (2020). Effects of Zishen Yutai Pills on ovarian reserve and pregnancy outcomes in infertile women. *Electronic Journal of Practical Gynecology and Endocrinology*.

[49] Yang R. (2020). Clinical observation of treating polycystic ovary syndrome with traditional Chinese medicine retention irrigation based on collateral disease theory. *Chinese Medicine Bulletin*.

[50] Wang C. (2019). The application of traditional Chinese medicine enema therapy in the treatment of gynecological diseases. *Jiangxi Chinese Medicine*.

[51] Wang T., Lu H., Li F., Zhang Q. (2021). Effect of Kangfuxin Liquid enema combined with mesalazine on gestational outcomes and quality of life in child-bearing female with active ulcerative colitis: a protocol for randomized, double-blind, controlled trial. *Medicine*.

[52] Duan H., Lu H (2010). Effect of formula for reinforcing kidney and activating blood on follicular development by rectal administration. *Chinese Materia Medica*.

[53] Zhao C. (2016). Effects of oral administration of traditional chinese medicine plus enema combined with HCG injection on ovulation in patients with LUFS Due to kidney deficiency and blood stasis. *Henan University of Traditional Chinese Medicine*.

[54] Liu H. (2016). Observation on the effect of clomiphene combined with Liuwei Dihuang Pill in the treatment of infertility with polycystic ovary syndrome. *Bethune Medical Journal*.

[55] Li Y. (2019). Effect of Zishen Yutai Pills on the efficacy of polycystic ovary syndrome combined with infertility and the effect of hormone levels. *Contemporary Medicine*.

[56] Zhao L., Xu Y., Duo X., Tian X., Wang P. (2018). A probe into effect of Shoutai pill on endometrial receptivity. *Experimental and Therapeutic Medicine*.

[57] Kunitomi C., Harada M., Kusamoto A. (2021). Induction of aryl hydrocarbon receptor in granulosa cells by endoplasmic reticulum stress contributes to pathology of polycystic ovary syndrome. *Molecular Human Reproduction*.

[58] Sørensen A. E., Udesen P. B., Wissing M. L., Englund A. L. M., Dalgaard L. T. (2016). MicroRNAs related to androgen metabolism and polycystic ovary syndrome. *Chemico-Biological Interactions*.

[59] Chen Y., Chai X., Zhao Y., Yang X., Zhong C., Feng Y. (2021). Investigation of the mechanism of zishen Yutai pills on polycystic ovary syndrome: a network pharmacology and molecular docking approach. *Evidence-based Complementary and Alternative Medicine*.

[60] Song J. Y., Gao D. D., Cao X. L. (2021). The role of traditional Chinese formula ding-kun pill (dkp) in expected poor ovarian response women (poseidon group 4) undergoing in vitro fertilization-embryo transfer: a multicenter, randomized, double-blind, placebo-controlled trial. *Frontiers in Endocrinology*.

[61] Xu Y., Guo Y., Song Y. (2018). A new theory for acupuncture: promoting robust regulation. *Journal of Acupuncture and Meridian Studies*.

[62] Ko J. H., Kim S. N. (2018). A literature review of women’s sex hormone changes by acupuncture treatment: analysis of human and animal studies. *Evidence-based Complementary and Alternative Medicine*.

[63] Wang Y. (2021). Clinical research progress of acupuncture in the treatment of infertility in the past 10 years. *Chinese Folk Therapy*.

[64] Quan K., Yu C., Wen X., Lin Q., Wang N., Ma H. (2022). Acupuncture as treatment for female infertility: a systematic review and meta-analysis of randomized controlled trials. *Evidence-based Complementary and Alternative Medicine*.

[65] Pan W., Li F., Wang Q. (2022). A randomized sham-controlled trial of manual acupuncture for infertile women with polycystic ovary syndrome. *Integrative Medicine Research*.

[66] Lai J. (2020). Clinical efficacy of clomiphene combined with traditional Chinese medicine and acupuncture in the treatment of polycystic ovary syndrome complicated with infertility. *Clinical Rational Drug Use*.

[67] Qiu X. (2019). Observation on the effect of acupuncture and moxibustion in the treatment of polycystic ovary syndrome complicated with infertility. *Collections*.

[68] Budihastuti U. R., Melinawati E., Sulistyowati S., Nurwati I. (2019). Electroacupuncture effect on polycystic ovary syndrome to improve oocytes’ growth. *Medical Acupuncture*.

[69] Kusuma A. C., Oktari N., Mihardja H. (2019). Electroacupuncture enhances number of mature oocytes and fertility rates for in vitro fertilization. *Medical Acupuncture*.

[70] Xie H. (2017). Study on the effect of kidney-tonifying, phlegm-removing and blood-activating method combined with acupuncture on endometrial receptivity in polycystic ovary syndrome. *Asia Pacific Traditional Medicine*.

[71] He X. (2021). Effect of drug combined with electroacupuncture on endometrial receptivity in infertile patients with polycystic ovary syndrome of kidney deficiency syndrome. *Chinese Journal of Traditional Chinese Medicine*.

[72] Li B. (2021). Analysis of the effect of traditional Chinese medicine Tiaojing Qutan prescription combined with electroacupuncture for kidney PCOS infertility patients. *Famous Doctors*.

[73] Budihastuti U. R., Melinawati E., Anggraini N. W. P. (2021). Electroacupuncture to improve endometrial receptivity and folliculogenesis in polycystic ovary syndrome. *Medical Acupuncture*.

[74] Peng Y., Guo L., Gu A. (2020). Electroacupuncture alleviates polycystic ovary syndrome-like symptoms through improving insulin resistance, mitochondrial dysfunction, and endoplasmic reticulum stress via enhancing autophagy in rats. *Molecular Medicine*.

[75] Xu K., Wang J., Hu F. (2021). Effects of moxibustion on reproduction and metabolism of polycystic ovary syndrome: a protocol for meta-analysis and systematic review. *BMJ Open*.

[76] Yu L. (2020). Effects of Yijing Yangshen Yunzi Prescription combined with moxibustion on sex hormone levels and ovarian hemodynamics in infertile patients with polycystic ovary syndrome and ovulatory disorder. *Hubei Journal of Traditional Chinese Medicine*.

[77] Liu S. (2020). Effects of warming acupuncture combined with Bushen Quyu Decoction on the prognosis of pregnancy and peripheral blood PAgT and D-D levels in infertility due to kidney deficiency and blood stasis. *Clinical Research*.

[78] Xu C. (2020). Efficacy analysis of Du Meridian warm acupuncture combined with clomiphene in the treatment of infertility caused by polycystic ovary syndrome. *Zhonghua Journal of Traditional Chinese Medicine*.

[79] Cai X. (2017). Observation on the curative effect of acupoint injection in the treatment of infertility with polycystic ovary syndrome. *Clinical Research in Traditional Chinese Medicine*.

[80] Wen H. (2020). Clinical study on the treatment of infertility caused by polycystic ovary syndrome of kidney deficiency and blood stasis type with traditional Chinese medicine for tonifying the kidney and activating blood circulation combined with acupoint injection. *Chinese Journal of Clinical Research*.

[81] Li C. (2020). Clinical study on auricular point sticking combined with acupuncture and hormonal therapy for irregular menstruation. *Shaanxi Traditional Chinese Medicine*.

[82] Shen Y. (2021). Research progress of acupuncture and moxibustion regulating the hypothalamic-pituitary-ovarian axis in the treatment of polycystic ovary syndrome. *China Journal of Integrative Medicine*.

[83] Shen L. (2022). Effects of electroacupuncture on the hypothalamic NF*κ*B pathway of pancreatic antibodies in rats with polycystic ovary syndrome. *Clinical Journal of Acupuncture and Moxibustion*.

[84] Zhu S., Wang Y., Chang X., Chen H., Jin X (2020). The protective effect of pre-moxibustion on reproductive hormones profile of rats with **<i>Tripterygium</i>**glycosides-induced ovarian damage. *Complementary medicine research*.

[85] Huang J., Tang C. L., Liao D. M. (2020). Effect of electroacupuncture on levels of serum sex hormones and expression of ovarian auto-phagy related factors in rats with polycystic ovary syndrome. *Zhen ci yan jiu Acupuncture Research*.

[86] Chen X., Tang H., Liang Y. (2021). Acupuncture regulates the autophagy of ovarian granulosa cells in polycystic ovarian syndrome ovulation disorder by inhibiting the PI3K/AKT/mTOR pathway through LncMEG3. *Biomedicine & Pharmacotherapy*.

[87] Ma T., Cui P., Tong X. (2018). Endogenous ovarian angiogenesis in polycystic ovary syndrome-like rats induced by low-frequency electro-acupuncture: the CLARITY three-dimensional approach. *International Journal of Molecular Sciences*.

[88] Yin Y., Zhang Y., Zhang H., Jiang D., Guo G. (2018). Clinical therapeutic effects of acupuncture combined with Chinese herbal medicine on infertility of polycystic ovary syndrome in the patients with ovulation induction with letrozole. *Zhongguo zhen jiu= Chinese acupuncture & moxibustion*.

[89] Xiang S., Xia M., Song J., Liu D., Lian F. (2021). Effect of electro-acupuncture on expression of IRS-1/PI3K/GLUT4 pathway in ovarian granulosa cells of infertile patients with polycystic ovary syndrome-insulin resistance of phlegm-dampness syndrome. *Chinese Journal of Integrative Medicine*.

[90] Li H. X., Shi L., Liang S. (2022). Moxibustion alleviates decreased ovarian reserve in rats by restoring the PI3K/AKT signaling pathway. *Journal of Integrative Medicine*.

[91] Lai M. H., Ma H. X., Li J., Song X. H., Liu H. (2016). Effects of electroacupuncture on mRNA expressions of insulin-receptor substrates 1 and 2 in the endometrium of PCOS rats and insulin sensitivity. *China Journal of Integrative Medicine*.

[92] Van Eekelen R., van Geloven N., van Wely M. (2019). IVF for unexplained subfertility; whom should we treat?. *Human Reproduction*.

[93] Xin X. (2022). Research progress on the application of electroacupuncture in the field of assisted reproduction in patients with polycystic ovary syndrome and related mechanisms. *Journal of Traditional Chinese Medicine*.

[94] Zhong Y., Zeng F., Liu W., Ma J., Guan Y., Song Y. (2019). Acupuncture in improving endometrial receptivity: a systematic review and meta-analysis. *BMC Complementary and Alternative Medicine*.

[95] Yuan L., Feng F., Mao Z. (2020). Effects of erbuzhuyu decoction combined with acupuncture on endometrial receptivity are associated with the expression of mir-494-3p. *Evidence-based Complementary and Alternative Medicine*.

[96] Chen W., Chen J., Xu M. (2019). Electroacupuncture facilitates implantation by enhancing endometrial angiogenesis in a rat model of ovarian hyperstimulation. *Biology of Reproduction*.

[97] Shen J., Chen L., Cheng J. (2019). Circular RNA sequencing reveals the molecular mechanism of the effects of acupuncture and moxibustion on endometrial receptivity in patients undergoing infertility treatment. *Molecular Medicine Reports*.

[98] Cena H., Chiovato L., Nappi R. E. (2020). Obesity, polycystic ovary syndrome, and infertility: a new avenue for GLP-1 receptor agonists. *Journal of Clinical Endocrinology and Metabolism*.

[99] Wu C. (2022). Weight management in polycystic ovary syndrome—aerobic exercise. *Open book is beneficial: Seeking Medical Advice*.

[100] Yang J., Chon T. Y., Bauer B. A. (2019). Use of acupuncture in overweight/obese women with polycystic ovary syndrome. *Medical Acupuncture*.

[101] Xu J., Zuo Y. (2018). Efficacy of acupuncture as adjunctive treatment on infertility patients with polycystic ovary syndrome. *Zhongguo zhen jiu= Chinese acupuncture & moxibustion*.

[102] Dou Z., Ma S. H., Song J. Y., Xia T. (2021). Retrospective analysis on pregnancy outcomes and fat-related factors of treatment of endomorph PCOS infertility patients by acupuncture of 8 acupoints around umbilicus. *Zhen ci yan jiu Acupuncture Research*.

[103] Shen L. Y., Liang C. M., Yang W. J., Pan L., Li H., Hu H. (2018). Acupuncture treatment of polycystic ovarian syndrome patients with abdominal obesity by regulating dai meridian: a randomized controlled clinical trial. *Zhen ci yan jiu= Acupuncture research*.

[104] Huang S., Hu M., Ng E. H. Y. (2020). A multicenter randomized trial of personalized acupuncture, fixed acupuncture, letrozole, and placebo letrozole on live birth in infertile women with polycystic ovary syndrome. *Trials*.

[105] Rooney K. L., Domar A. D. (2022). The relationship between stress and infertility. *Dialogues in Clinical Neuroscience*.

[106] Tan A., Wang M., Liu J. (2020). Efficacy and safety of acupuncture combined with western medicine for anxiety: a systematic review protocol. *Medicine*.

[107] Wang Z., Dong H., Wang Q. (2019). Effects of electroacupuncture on anxiety and depression in unmarried patients with polycystic ovarian syndrome: secondary analysis of a pilot randomised controlled trial. *Acupuncture in Medicine*.

[108] Sabbagh Gol A., Rezaei Ardani A., Farahmand S. K. (2021). Additive effects of acupuncture in alleviating anxiety: a double-blind, three-arm, randomized clinical trial. *Complementary Therapies in Clinical Practice*.

[109] Kim Y. J., Kim S. H., Lee H. J., Kim W. Y. (2018). Infectious adverse events following acupuncture: clinical progress and microbiological etiology. *Journal of Korean Medical Science*.

[110] Witt C. M., Pach D., Brinkhaus B. (2009). Safety of acupuncture: results of a prospective observational study with 229, 230 patients and introduction of a medical information and consent form. *Complementary Medicine Research*.

[111] Moon H. Y., Kim M., Hwang D. (2020). Safety of acupuncture during pregnancy: a retrospective cohort study in Korea. *BJOG: An International Journal of Obstetrics and Gynaecology*.

[112] Park J., Sohn Y., White A. R., Lee H. (2014). The safety of acupuncture during pregnancy: a systematic review. *Acupuncture in Medicine*.

[113] Dubey P., Reddy S., Boyd S. (2021). Effect of nutritional supplementation on oxidative stress and hormonal and lipid profiles in PCOS-affected females. *Nutrients*.

[114] Butts S. F., Seifer D. B., Koelper N. (2019). Vitamin D deficiency is associated with poor ovarian stimulation outcome in PCOS but not unexplained infertility. *Journal of Clinical Endocrinology and Metabolism*.

[115] Lerchbaum E., Theiler-Schwetz V., Kollmann M. (2021). Effects of vitamin D supplementation on surrogate markers of fertility in pcos women: a randomized controlled trial. *Nutrients*.

[116] Guo S., Tal R., Jiang H., Yuan T., Liu Y. (2020). Vitamin D supplementation ameliorates metabolic dysfunction in patients with PCOS: a systematicreview of RCTs and insight into the underlying mechanism. *International Journal of Endocrinology*.

[117] Snider A. P., Wood J. R. (2019). Obesity induces ovarian inflammation and reduces oocyte quality. *Reproduction*.

[118] Zhao J. F., Li B. X., Zhang Q. (2021). Vitamin D improves levels of hormonal, oxidative stress and inflammatory parameters in polycystic ovary syndrome: a meta-analysis study. *Annals of Palliative Medicine*.

[119] Chen J., Guo Q., Pei Y. (2020). Effect of a short-term vitamin E supplementation on oxidative stress in infertile PCOS women under ovulation induction: a retrospective cohort study. *BMC Women’s Health*.

[120] Szczuko M., Hawrylkowicz V., Kikut J., Drozd A. (2020). The implications of vitamin content in the plasma in reference to the parameters of carbohydrate metabolism and hormone and lipid profiles in PCOS. *The Journal of Steroid Biochemistry and Molecular Biology*.

[121] Yu G. (2020). A clinical study on the effect of supplementation of trace elements on promoting ovulation in infertile patients with polycystic ovary syndrome. *China Modern Drug Application*.

[122] Yu Q. (2021). Chinese expert consensus on reproductive health and multiple micronutrient supplementation. *Chinese Journal of Practical Gynecology and Obstetrics*.

[123] Montanino Oliva M., Zuev V., Lippa A., Carra M. C., Lisi F. (2019). Efficacy of the synergic action of myoinositol, tyrosine, selenium and chromium in women with PCOS. *European Review for Medical and Pharmacological Sciences*.

[124] Zhen J. (2018). Endocrinology group and guideline expert group of Chinese Medical Association Obstetrics and Gynecology Branch, Chinese guidelines for diagnosis and treatment of polycystic ovary syndrome. *Chinese Journal of Obstetrics and Gynecology*.

[125] Zhang H. (2022). Study on the effect and compliance of weight management with CRD in obese patients with PCOS. *Chongqing Medicine*.

[126] Gou Y. (2022). Research progress on the etiology, pathogenesis and treatment of polycystic ovary syndrome. *Journal of Practical Chinese Medicine*.

[127] Barrea L., Marzullo P., Muscogiuri G. (2018). Source and amount of carbohydrate in the diet and inflammation in women with polycystic ovary syndrome. *Nutrition Research Reviews*.

[128] Huang F. (2021). Effects of weight management on metabolism and pregnancy outcomes in overweight and obese PCOS patients. *Chinese Journal of Family Planning*.

[129] Paoli A., Mancin L., Giacona M. C., Bianco A., Caprio M. (2020). Effects of a ketogenic diet in overweight women with polycystic ovary syndrome. *Journal of Translational Medicine*.

[130] Tan L. (2021). The effect of different weight management methods on the outcome of in vitro fertilization-embryo transfer in overweight or obese polycystic ovary syndrome.

[131] King A. K., McGill M., Beller, Solorzano B. (2019). Go Girls!—dance-based fitness to increase enjoyment of exercise in girls at risk for PCOS. *Children*.

[132] Li N., Yang C., Xie H., Liu Y., Liao Y. (2021). Effects of aerobic exercise on rats with hyperandrogenic polycystic ovarian syndrome. *International Journal of Endocrinology*.

[133] Hakimi O., Cameron L. C. (2017). Effect of exercise on ovulation: a systematic review. *Sports Medicine*.

[134] Richards C. T., Meah V. L., James P. E., Rees D. A., Lord R. N. (2021). HIIT’ing or MISS’ing the optimal management of polycystic ovary syndrome: a systematic review and meta-analysis of high-versusmoderate-intensity exercise prescription. *Frontiers in Physiology*.

[135] Wang L., Liu J., Li Y. (2021). Modified Nuss operation using introducer-bar complex for pectus excavatum in adults: a retrospective study. *Journal of Cardiothoracic Surgery*.

[136] Peng J. (2021). Observation on the clinical efficacy of kidney-tonifying and phlegm-relieving formula combined with Taijiquan exercise on knee osteoarthritis due to kidney deficiency and phlegm turbidity. *Journal of Tianjin University of Traditional Chinese Medicine*.

[137] Tian G. (2022). Analysis of the incidence of depression and anxiety and its influencing factors in PCOS infertility patients_Tian Guohua. *Clinical Medicine Research and Practice*.

[138] Mohseni M., Eghbali M., Bahrami H., Dastaran F., Amini L. (2021). Yoga effects on anthropometric indices and polycystic ovary syndrome symptoms in women undergoing infertility treatment: a randomized controlled clinical trial. *Evidence-based Complementary and Alternative Medicine*.

[139] Sun X. (2019). Analysis of the current status of health-related quality of life in patients with polycystic ovary syndrome of reproductive age. *Journal of Reproductive Medicine*.

[140] Li L., Ning N., Wei J. (2021). Metabonomics study on the infertility treated with zishen Yutai pills combined with in vitro fertilization-embryo transfer. *Frontiers in Pharmacology*.

[141] Wang L., Yu C. C., Li J., Tian Q., Du Y. J. (2021). Mechanism of action of acupuncture in obesity: a perspective from the hypothalamus. *Frontiers in Endocrinology*.

[142] Li J. (2020). Effects of cognitive-behavioral interventions on dysphoria and pregnancy outcomes in patients with PCOS infertility. *Psychological Monthly*.

